# Ultra-Liquid Chromatography Tandem Mass Spectrometry (UPLC-MS/MS)-Based Pharmacokinetics and Tissue Distribution Study of Koumine and the Detoxification Mechanism of *Glycyrrhiza uralensis* Fisch on *Gelsemium elegans* Benth.

**DOI:** 10.3390/molecules23071693

**Published:** 2018-07-11

**Authors:** Lin Wang, Qi Sun, Nan Zhao, Yan-Qing Wen, Yang Song, Fan-Hao Meng

**Affiliations:** School of Pharmacy, China Medical University, 77 Puhe Road, Shenyang 110122, Liaoning, China; wanglin@cmu.edu.cn (L.W.); zgydsunqi@163.com (Q.S.); zhaonan-dd@163.com (N.Z.); cmu20038915@sina.com (Y.-Q.W.); Songyanglhyb1998@163.com (Y.S.)

**Keywords:** *Gelsemium elegans* Benth., *Glycyrrhiza uralensis* Fisch, koumine, pharmacokinetics, tandem mass spectrometry, detoxification

## Abstract

*Gelsemium elegans* Benth. (*G. elegans*), which is a famous Chinese folk medicine, has been commonly used to treat certain types of skin ulcers and alleviate inflammation, headaches, and cancer pain. However, the extensive clinical use of *G. elegans* has been greatly hampered by its toxicity. As one of the most widely used herbal medicines, *Glycyrrhiza uralensis* Fisch, has a unique effect on detoxification of *G. elegans*. In the present study, a rapid and sensitive method using ultra-liquid chromatography tandem mass spectrometry (UPLC-MS/MS) was established and validated for determination of koumine, the most abundant molecule among the alkaloids of *G. elegans*, in rat plasma, tissue, and liver microsome. The developed method was successfully applied to the pharmacokinetics, tissue distribution, and in vitro metabolism study in rat with or without pre-treated *Glycyrrhiza uralensis* Fisch extract. Meanwhile, the expression level of CYP3A1 mRNA was analyzed to explain the detoxification mechanism of *Glycyrrhiza uralensis* Fisch on *G. elegans.* As a result, our work demonstrated that *Glycyrrhiza uralensis* Fisch could significantly affect the pharmacokinetics and tissue distribution of koumine in rats. The detoxification mechanism of *Glycyrrhiza uralensis* Fisch on *G. elegans* may be its cytochrome enzyme up-regulation effect.

## 1. Introduction

*Gelsemium elegans* Benth. (*G. elegans*) is distributed in China and Southeastern Asia. It was widely used as a classic Chinese herbal medicine to treat malignant skin problems [1]. Recent years, its anti-tumor [2], anti-inflammatory [3], and anxiolytic [4] activities have also been well studied. On the other side, *G. elegans* was well-known for its toxicity. Typical symptoms of intoxication include vomiting, blurred vision, muscular weakness, limb paralysis, dilated pupils, breathing difficulty, and convulsion. In instances of severe poisoning, the nervous system is depressed and death is caused by respiratory depression [5,6]. Its toxicities set a limit to the application of *G. elegans* in clinical settings. It is critical finding effective methods to reduce the toxicity of *G. elegans* based on the mechanism of action. 

*Glycyrrhiza uralensis* Fisch (GU) is one of the most widely used herbal medicines. According to traditional Chinese medicine (TCM) theory, GU is primarily effective for fatigue and debilitation, asthma with coughing, and excessive phlegm. Moreover, it has a unique effect on moderating the characteristics of toxic herbs, which could be partly interpreted as detoxification [7]. In the folk, intragastric administration of GU was used to detoxify *G. elegans*, much of the work carried out on the role of GU on CYPs regulation and function, though further investigation was required for the mechanism of this detoxification method. 

As the most abundant molecule among the alkaloids of *G. elegans*, koumine (KM) has been demonstrated to exert numerous potent biological properties, such as anxiolytic and analgesic effects [8]. However, it also possesses inhibitory effects on splenocyte proliferation and the humoral immune response [9]. In the previous study, our group reported the pharmacokinetics study of gelsemine and koumine after oral administration of the extract of *G. elegans* [10], but the tissue distribution data in the literature is limited. Furthermore, there is no report about the influence of GU on the pharmacokinetics and tissue distribution of KM and we speculate that GU may have an impact on the pharmacokinetics of KM metabolized by inducing the CYP450 system. The aim of this work is to evaluate the pharmacokinetics and tissue distribution properties of KM in rats and to explore how these behaviors are altered by the pre-treated GU extract. It desrved further investigation so as to understand the possibility regarding the combination use of KM and GU. To achieve this, an ultra-liquid chromatography tandem mass spectrometry (UPLC-MS/MS) method was developed and validated for the determination of koumine in rat plasma, tissues, and rat liver microsome (RLM). In vivo pharmacokinetic and tissue distribution study could give a straightforward result for the influence of GU on KM. Moreover, GU is discussed for testing its potential on hepatic enzyme inductions by both in vitro metabolism and RT-qPCR study. The results of our study would provide a meaningful basis for evaluating the rationality for the detoxification effect of GU on *G. elegans*.

## 2. Results

### 2.1. Method Development

Liquid-liquid extraction (LLE) and the protein precipitation method (PPM) were compared during sample preparation. PPM, which is a simple and fast technique, has obtained satisfactory recoveries and reduced the endogenous-related substances in bio-matrix. The recovery data for both LLE and PPM was shown in supporting information Table S1.

**Figure 1:**
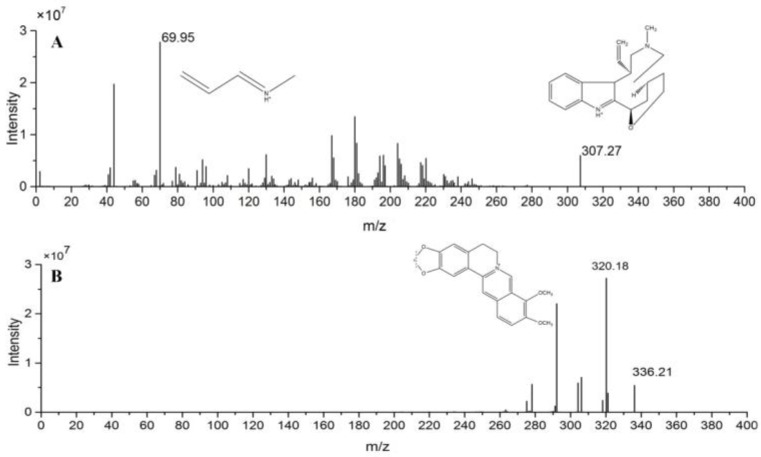
Chemical structures and full scan product ion of precursor ions of koumine (KM) (**A**) and IS (**B**).

Both KM and internal standard(IS) exhibited higher sensitivity in the positive mode than in the negative mode. Quantification was performed using the multiple reaction monitoring (MRM) mode of the transitions *m*/*z* [M + H]^+^ 307.1→70.8 for KM and *m*/*z* [M + H]^+^ 336.0→320.4 for IS. The mass spectrometer was operated at collision energy of 25 and 15 V for KM and IS, respectively, capillary voltage of 2.0 kV, cone voltage of 44 V, source temperature of 120 °C, desolvation temperature 500 °C, and desolvation gas 1000 L/h. The ion pairs were selected for the final MRM method is given in Figure 1.

### 2.2. Method Validation

#### 2.2.1. Specificity

**Figure 2:**
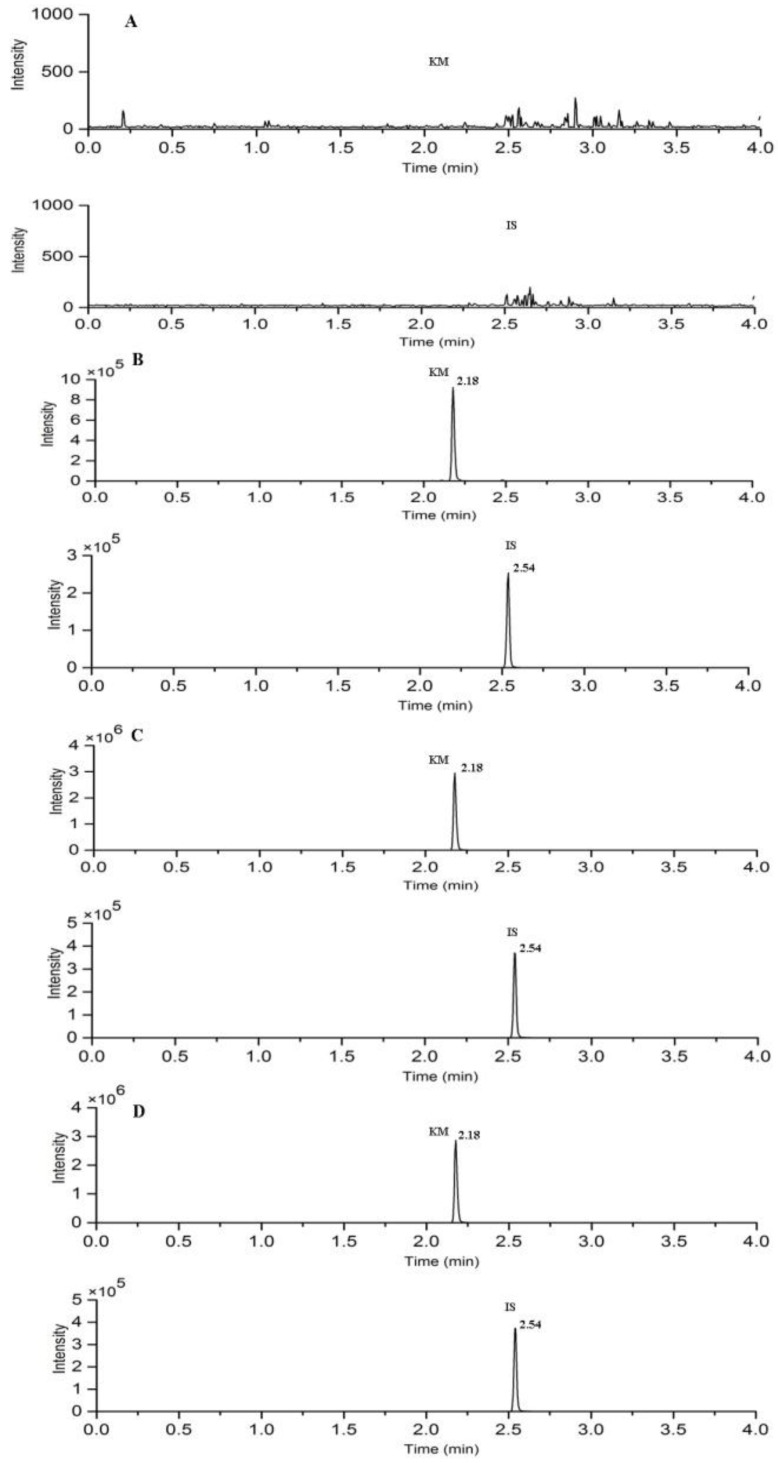
Typical chromatograms of (**A**) blank rat plasma; (**B**) blank rat plasma spiked with KM and IS; (**C**) rat plasma sample at 20 min; and, (**D**) liver sample at 30 min after a single intravenous dose of 10 mg/kg KM. Representative MRM chromatograms of KM and IS.

Under the UPLC-MS/MS conditions that are described above, the retention time of KM and IS were 2.18 and 2.54 min, respectively. The typical chromatograms of blank plasma, blank plasma spiked with the analytes and IS, plasma collected at 20 min, and liver sample at 30 min after intravenous administration of KM are shown in Figure 2. No significant endogenous interference was found in the elution time of analyte and IS. 

#### 2.2.2. Linearity and Lower Limit of Quantification LLOQ

Calibration curves were constructed for KM and IS by least-squares linear calibration. Moreover, the present UPLC-MS/MS method that is offered LLOQ values in rat plasma and various tissues less than 25 ng/mL. The regression equation, correlation, and linear ranges are shown in Table 1, which indicated that the method was sensitive enough for the pharmacokinetics, tissue distribution, and in vitro metabolism study.

#### 2.2.3. Precision and Accuracy

The precision and accuracy were estimated by analyzing QC samples in six replicates. The intra-day precision was determined on the same day and the inter-day precision was determined on three consecutive days. Precisions were expressed as RSD required less than 15%, and the accuracy to be within ±15%. The intra- and inter-day precisions were less than 12.11%, and the accuracy ranged from −4.32% to 4.51%. The data in all of the biological matrices of rats were summarized in Table 2, which demonstrated that this assay was accurate and reproducible for the determination of KM in rat plasma, tissues, and RLM.

#### 2.2.4. Extraction Recovery and Matrix Effect

The matrix effects were determined by six independent sources of matrix. It was calculated by comparing the responses of the post extracted biological samples with that of pure standard solution containing equivalent amounts of the analytes. The extraction recovery was performed by comparing the peak area rations of KM to IS of an extracted sample to the standard analytes solution of the same concentration.

Extraction recovery for KM and IS in all of the biological matrices were greater than 78.58%. In addition, the matrix effect values ranged from 84.32 to 105.12% for KM. These results suggested that the evaluated method was free of matrix effects and reliable for bioanalysis. The results are shown in Table 3.

#### 2.2.5. Stability

The results for short-term stability, three freeze-thaw cycles and long term stability are summarized in Table 4. It is illustrated that KM was stable enough in bio-matrix.

### 2.3. Pharmacokinetics Study and Tissue Distribution

**Figure 3:**
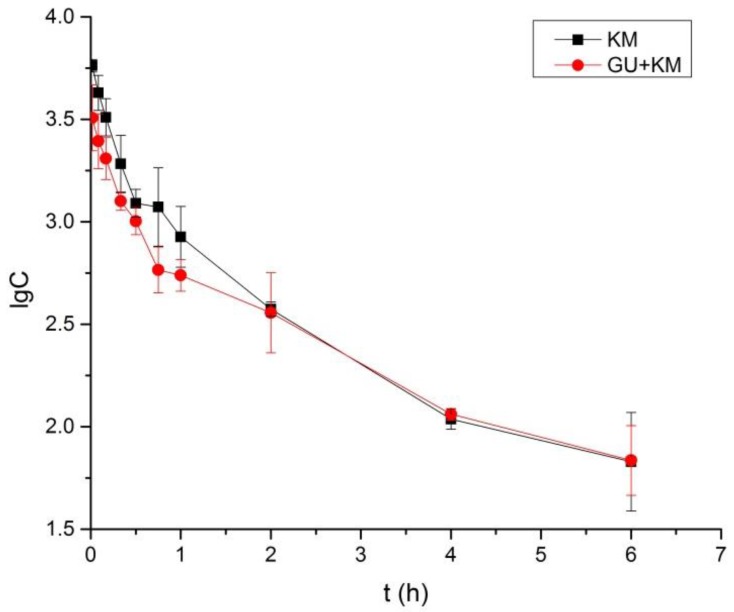
Mean ± SD (n = 6) plasma concentration-time profiles of koumine following intravenous administration of 10 mg/kg KM that received or did not receive *Glycyrrhiza uralensis* Fisch (GU) (3 g/Kg, once daily for 14 days).

The pharmacokinetics parameters, including the areas under concentration-time curve (AUC_0–6_ and AUC_0–∞_), mean retention time (MRT_0–t_ and MRT_0–∞_), the half-time (*t*_1/2z_), clearance (CL_Z_), apparent volume of distribution (V_z_), and maximum plasma concentration (*C*_max_) were presented in Table 5. The parameters of KM in the GU + KM group, such as *C*_max_, *t*_1/2z_, AUC_0__–6_, AUC_0__–∞_, and CL_Z_ statistically differed from those in control group (The *p*-values for *C*_max_, *t*_1/2z_, AUC_0–6_, AUC_0__–__∞_, CL_Z_, and V*_Z_* were 0.015, 0.035, 0.019, 0.039, and 0.029, *p* < 0.05.). Moreover, notably, the V_Z_ showed significant differences between the two groups (The *p*-values for V*_Z_* was 0.002, *p* < 0.01). The results implied that GU may have effects on reducing the exposure of KM. The mean plasma concentration-time profiles were shown in Figure 3.

**Figure 4:**
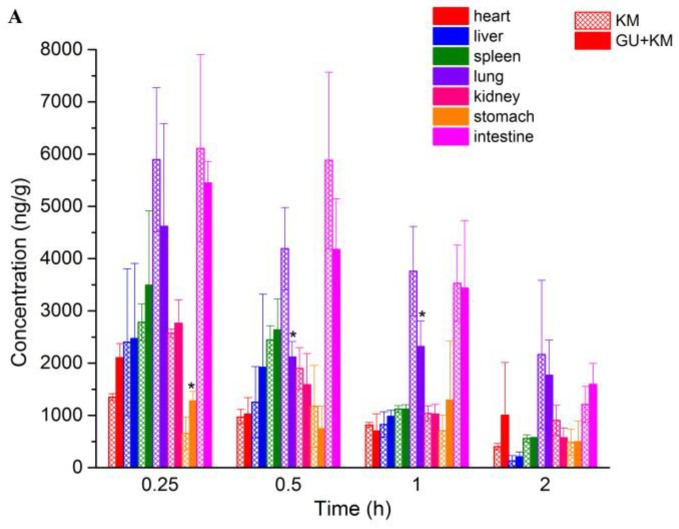

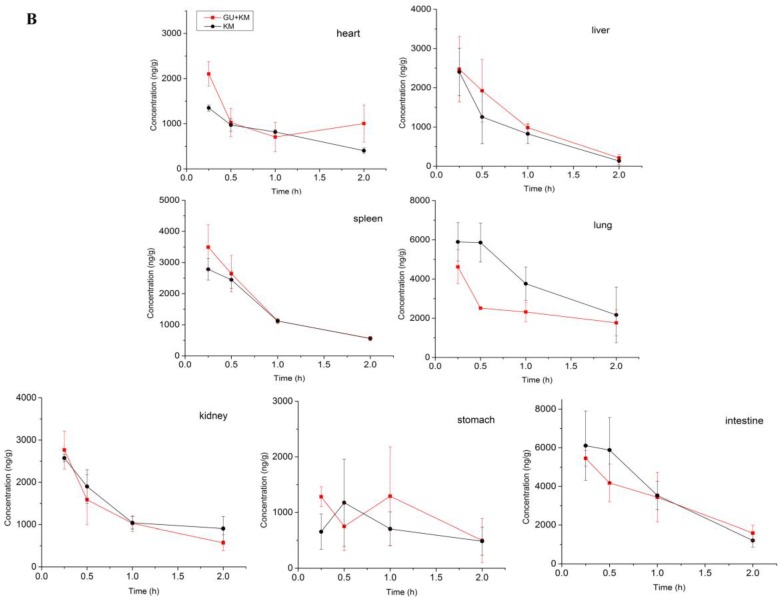
Mean ± SD (n = 6) concentration-time profiles (**A**) and distribution decline trend (**B**) of koumine in tissues following intravenous administration of 10 mg/kg KM that received or did not receive GU (3 g/Kg, once daily for 14 days, * *p* < 0.05, compared with the KM group).

The concentrations of KM in various tissues for both groups are shown in Figure 4A. At 15 min after intravenous injection, different concentrations of KM were detected in all of the rat tissues. It showed that KM could be distributed widely and rapidly in various tissues. With the extent of time, the concentrations of KM in most of the tissues decreased obviously in 2 h. This indicated that there was no long-term accumulation of KM, which was in accordance with the change of plasma concentration. For the control group, the highest concentration level was observed in intestine (6109.35 ± 1795.14 ng/g), followed by lung (5893.27 ± 1383.72 ng/g), spleen (2781.9 ± 348.6 ng/g), kidney (2571.9 ± 82.2 ng/g), liver (2402.2 ± 1406.0 ng/g) heart (1623.2 ± 477.8 ng/g), and stomach (655.6 ± 315.6 ng/g), which implied that intestine and lung might be the target organs of KM. That is probably the pharmacokinetics basis of KM for good therapeutic effect on digestive system tumors [11]. The higher concentration of kidney and liver demonstrated that KM was mainly accumulated in liver and renal excretion might be a main elimination route for KM. In addition, there was a statistic difference for the tissue concentration for lung and stomach between the two groups (*p* < 0.05. The *p* values were 0.041, 0.037, and 0.014 for stomach at 0.25 h, and lung at 0.5 h and 1 h, respectively). Moreover, it is showed in Figure 4B that concentration of KM declined more sharply for the GU + KM group than the control group. These results give a hint that GU may affect the pharmacokinetics and tissue distribution characteristics of KM.

### 2.4. In Vitro Metabolism of Koumine in Rat Liver Microsomes

**Figure 5:**
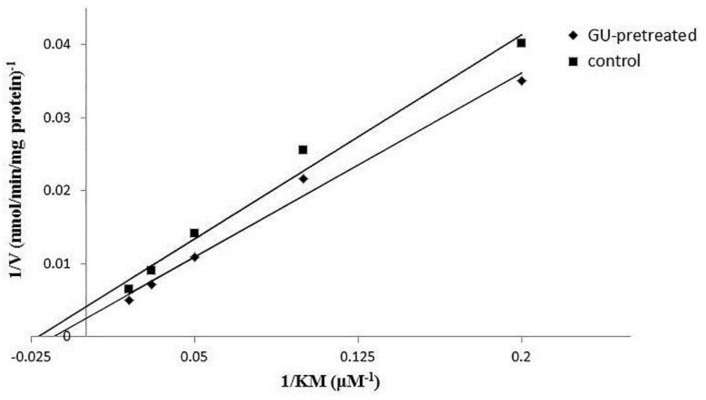
Lineweaver-Burk plot of 5, 10, 20, 30, and 50 μm KM that received or did not receive GU following 30 min of incubation.

Michaeli-Menten constant (*K_m_*) and maximum velocity (*V_max_*), the incubation experiments were carried out to estimate apparent kinetics parameters by different concentration of KM. According to Michaeli-Menten equation, the velocity of CYP3A1-catalyzed reaction was plotted as a function of KM concentration. Figure 5 showed the plot of *1*/*V* vs. *1*/*S* (1/KM), and the corresponding Lineweaver-Burk plot. By fitting the data with Michaelis-Menten equation, corresponding *K_m_*, *V_max_,* and CL_int_ for the metabolism mediated by CYP3A1 were determined and showed in Table 6. There was a statistic difference for *V_max_* between the two groups (*p* < 0.05). It can be inferred that GU may alleviate the toxicity by accelerating the metabolism of KM.

### 2.5. Effects of GU Treatment on CYP3A1 Expression by RT-qPCR

**Figure 6:**
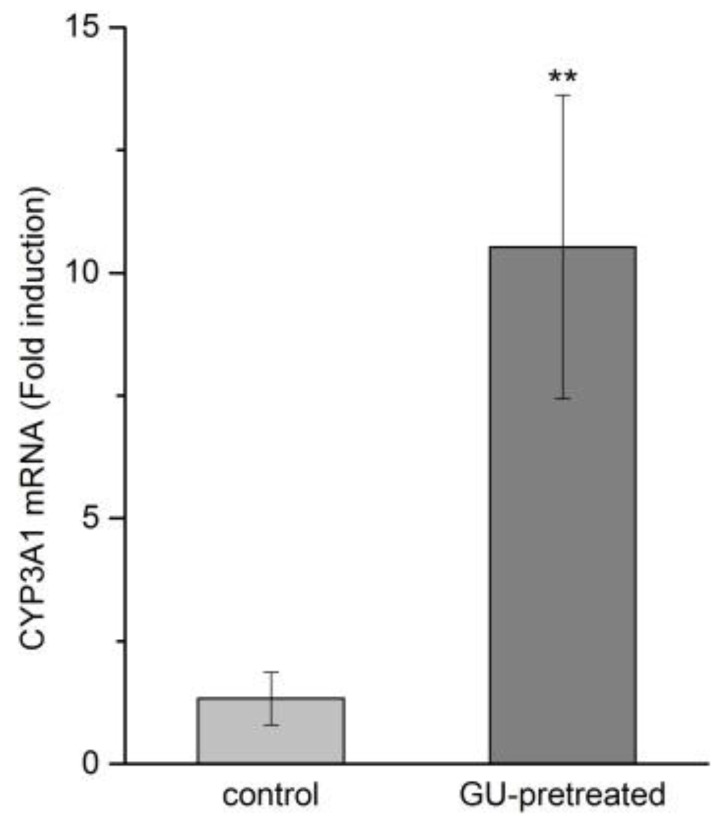
Effect of GU on the induction of CYP3A1 mRNA expression (n = 5). Data are expressed as mean ± SD. ** *p* < 0.05 compared with the control group.

GU possesses a remarkable detoxifying activity that can treat drug and food poisoning or inhibit adverse effects. The exact mechanism of this effect is not well identified, but is believed to be related to its regulation of cytochrome P450 (CYP450) enzymes. Studies have indicated KM was metabolized by the CYP3A1, which is the main isoforms of cytochrome P450 in rat [12]. In this study, RT-qPCR was performed in order to investigate whether the GU treatment could induce the CYP3A1 expression. Treatment with GU resulted in a marked induction of the mRNA expression of CYP3A1 in liver tissue when compared to the control group (Figure 6, 8-fold, *p* < 0.01). It can be illustrated that GU tended to increase the mRNA expression level of CYP3A1, which is the major metabolic enzyme that is involved in KM metabolism. 

## 3. Experimental

### 3.1. Materials and Reagents

Standard of KM (98% purity) were purchased from Qingdao JieShiKang Biotechnology (Qingdao, Shandong, China). The berberine (internal standards, I.S., 98% purity) was provided from National Institute for the Control of Pharmaceutical and Biological Products (0713-9906, Beijing, China). The NADPH-regenerating system containing glucose 6-phosphate (G-6-P), glucose-6-phosphate dehydrogenase (G-6-PDH), NADPH^+^, and MgCl_2_ was purchased premixed from BD Biosciences (San Jose, CA, USA). HPLC-grade acetonitrile was purchased from Merck (Merck, Darmstadt, Germany). Deionized water was produced from a Milli-Q water purification system (Millipore, Billerica, MA, USA). Other reagents were of analytical grade. T10 basic ULTRA-TURRAX homogenizer (IKA company, Staufen, Germany).

Pharmacokinetic parameters were estimated using the drug and statistic (DAS) software. Statistical analysis was used the IBM spss statistics software and an independent-sample t test.

*Glycyrrhiza uralensis* Fisch was collected from the Bozhou Traditional Chinese Medicine Market of Anhui and identified by Professor Dongfang Zhang of China Medical University. 

### 3.2. Animals

Male adult Sprague-Dawley rats (180–220 g) were supplied by the Experiment Animal Center, China Medical University. The experimental protocol was approved by the Animal Ethics Committee of China Medical University (Permit number: SYK2017–0019), and all animal studies were carried out according to the Guide for Care and Use of Laboratory Animals.

### 3.3. Preparation of Herbal Decoctions

The powdered GU was added to fourfold of distilled water and immersed for 30 min at room temperature. The mixture was heated at reflux for 1 h. The filtrates were extracted again in the same way. The combined extract solution was evaporated under reduced pressure to yield a dark brown residue. The extraction yield was 32.6%. For administration to the animals, the dose was calculated on the base of original herbs. Dried residues were reconstituted with distilled water and adjusted to such a volume that 1 mL of the final decoction contained 1 g of equivalent original herbal material.

### 3.4. Preparation of Calibration Standards and Quality Control (QC) Samples

The stock solution of KM (0.5 mg/mL) and IS (1 μg/mL) were separately prepared in methanol. The stock solution of KM was further diluted into a series of working standard solutions with methanol. The calibration standard solution were prepared by spiking 10 μL standard working solutions, which was evaporated to dryness by a gentle stream of nitrogen and then mixed with 100 μL blank biological matrix to yield calibration concentrations of 10–5000 ng/mL for plasma, 25–5000 ng/mL for tissues, and 50–50000 ng/mL for RLM. QC samples at low, middle, and high concentrations were prepared at 25 ng/mL, 300 ng/mL, and 4000 ng/mL for plasma, 100 ng/mL, 600 ng/mL, and 4000 ng/mL for tissues and 250 ng/mL, 2000 ng/mL, and 20,000 ng/mL for RLM.

### 3.5. Sample Preparation

An aliquot of 10 μL IS solution was added to 100 μL biological matrix. The mixture was then precipitated with 300 μL methanol. The mixture was vortexed for 1 min and centrifuged at 12,000 rpm for 15 min. The supernatant was transferred to a clean tube and dried under nitrogen gas. The residue was reconstituted with 50 μL methanol, after being centrifuged at 15,000 rpm for another 15 min, 5 μL of the sample solution was injected into the UPLC-MS/MS system for analysis.

Each weighed tissue sample was thawed and then homogenized in ice-cold physiological saline (1:2, *w*/*v*). Subsequent steps were identical to those that are described above.

### 3.6. Instruments and Analytical Conditions

An ACQUITY UPLC system (Waters Corp. Milford, MA, USA), equipped with cooling auto-sampler and column oven enabling temperature control was used for all analysis. Chromatographic separation was performed on an ACQUITY UPLC^®^ BEH C_18_ column (3.0 × 50 mm, 1.7 µm) and the column temperature was maintained at 25 °C. The mobile phase consisted of acetonitrile (A) and water containing 0.05% formic acid (B). The gradient elution program was as follows: 0–0.5 min, 5% A, 0.5–2.1 min, 70% A, 2.1–3.0 min, 5% A. The flow rate was 0.4 mL/min and the injection volume was 5 µL. 

Mass spectrometry was performed on a Waters Xevo TQD tandem quadrupole mass spectrometers (Waters Corp., Milford, MA, USA) equipped with an electrospray ionization (ESI) interface. The instrument was operated in positive ion MRM mode under the following setting parameters: capillary voltage 2.0 kV, cone voltage 44 V, source temperature 120 °C, desolvation temperature 500 °C, and desolvation gas 1000 L/h. 

### 3.7. Method Validation

The method validation was fully conducted according to the guidelines of the Food and Drug Administration (FDA) for evaluating the specificity, linearity, precisions, accuracy, dilution integrity, extraction recovery, matrix effects, and stability. The method validation was seen supporting information “method validation” section. 

### 3.8. In Vivo Pharmacokinetics and Tissue Distribution Study

12 male SD rats were randomly divided into two groups (n = 6 per group), one as the single-dose of KM group, and the other as the repeated oral pre-treatment GU (GU + KM) group. Rats in the GU + KM group were administered GU decoction 3 g/kg by gavage once daily for 14 consecutive days. After 1 h of the last dosing, rats of the two groups were intravenously administered with 10 mg/kg KM. Blood sample (each approximately 0.3 mL) were collected into heprin sodium containing Eppendorf tubes from the suborbital vein at 0.017, 0.083, 0.17, 0.33, 0.50, 0.75, 1, 2, 4, 6 h post dose. The blood samples were immediately centrifuged at 4000 rpm for 10 min to collect plasma, which were then stored at −20 °C until further analysis by UPLC-MS/MS. Pharmacokinetics parameters were estimated by drug and statistic (DAS) software (version 3.0, Mathematical Pharmacology Professional Committee of China, Shanghai, China).

48 male SD rats were randomly divided into two groups (n = 24 per group), as above. After intravenous administration of KM, each group were further divided into four groups (n = 6 per group). To each group, heart, liver, spleen, lung, kidney, stomach and intestine were collected at 0.25, 0.5, 1 and 2 h. Tissue samples were rinsed with normal saline solution to remove the blood and were then weighed the wet weight and stored at −20 °C until further analysis by UPLC-MS/MS.

### 3.9. Preparation of RLM

Male SD rats were randomly divided into two groups of five animals each. The rats of GU-pretreated group received the decoction orally at a dose of 3 g/kg once daily for 14 days as well as the control group rats were given water only. On the day 15, rats were killed and the liver was rinsed in situ with 1.17% KCl via the hepatic portal vein and the dorsal aorta. The livers were removed immediately and then homogenized with buffer A (50 mM Tris-HCl buffer at pH 7.4 containing 0.2 M sucrose, 1:3, *w*/*v*). After the first centrifugation at 12,500× *g* for 30 min, the supernatant was further centrifuged at 100,000× *g* for 60 min at 4 °C. The microsomal pellets were suspended in buffer A with 20% glycerol. The microsomal protein content was quantified according to the method of Lowry et al. [13]. 

### 3.10. In Vitro Metabolism of Koumine in RLM

Microsomes prepared from both the GU-pretreated and control groups were used to assess *in vitro* the metabolism of KM. The NADPH-regenerating system contained 1 mg/mL microsomal proteins, 1.1 mM NADPH^+^, 0.035 mM MgCl_2_, 2.34 mM G-6-P, 0.28 U/mL G-6-P dehydrogenase, serial concentration of koumine (final concentration 5–50 μM) in phosphate buffer (pH 7.4, 0.1 M) in a total volume of 200 μL. The mixture was incubated at 37 °C for 30 min based on our preliminary studies to ensure the linear metabolic clearance rate of KM. The reactions were terminated with an equal volume of ice-cold acetonitrile, and then the samples were analyzed by UPLC-MS/MS. The equation of KM reaction velocity (*V*) in liver microsomes was expressed as *V* = (*C_0_* − *C_t_*)/*T*/*C_p_*, where *C_0_* and *C_t_* represent the initial concentration and the final concentration of KM in incubation solution, respectively, *T* is the reaction time (min) and *C_p_* is the protein concentration (mg/mL). All of the values were expressed as mean ± SD (*n* = 5). The mean intrinsic clearance rate (*CL_int_*) for the in vitro incubation was estimated by *V_max_*/*K_m_*.

### 3.11. RNA Isolation, cDNA Synthesis and Reverse Transcription-Quantitative Polymerase Chain Reaction (RT-qPCR) Analysis of CYP3A1 mRNA

Approximately 100–200 mg of liver tissue obtained from the rats of both the GU-pretreated and control groups. The liver tissue was homogenized and total RNA was extracted using the Trizol reagent, following the protocol. The RNA concentration was determined by a NANOROP 2000 spectrophotometer (Thermo Fisher Scientific Inc., Waltham, MA, USA). 1 mg of total RNA of each sample was reverse transcribed by the ReverTra Ace qPCR RT kit (TOYOBO CO., LTD., Osaka, Japan), according to the manufacture’s manual. All of the templates were diluted 10-fold prior to use in RT-qPCR. The expression level of CYP3A1 was determined in 96-well plates by an Mx3000P PCR system (Agilent Technologies Inc., Santa Clara, CA, USA). All of the PCR reactions was carried out using SYBR^®^ Green Realtime PCR Master Mix kit in a 13.5 μL reaction system containing 0.25 μL of each primer and 2 μL cDNA, 4.5 μL dd H_2_O, and 6.25 μL Ultra SYBR Mixture with ROX (TOYOBO CO., LTD.), following the manufacturer instructions. Primer sequences were as follows: for GAPDH, Forward primer: 5′-AGCCTCGTCCCGTAGACAAAA-3′, Reverse primer: 5′-TGGCAACAATCTCCACTTTGC-3′, for CYP3A4, Forward primer: 5′-TTCCCTCAACAACCCGAAGG-3′, Reverse primer: 5′-CTGCCCTTGTTCTCCTTGCT-3′. The reaction mixture was initially incubated at 95 °C for 2 min to denature DNA. Amplification was performed for 40 cycles of 94 °C for 30 s and 55 °C for 30 s and 72 °C for 30 s, and 72 °C for 5 min. 

## 4. Conclusions

A sensitive, rapid and specified UPLC-MS/MS method was established and successfully applied to the comparative pharmacokinetics and tissue distribution study of koumine in rats across different administrations. Moreover, by using this method, we compared the *in vitro* metabolism of koumine after pre-treated GU extract. The results indicated that *Glycyrrhiza uralensis* Fisch could significantly affect the pharmacokinetics and tissue distribution of koumine in rats. The mechanism of this behavior may be accounted for by the up-regulation of cytochrome enzyme by *Glycyrrhiza uralensis* Fisch. 

## Figures and Tables

**Table 1:** The regression equations, linear ranges, and LLOQs for the determination of the analytes in rat biological samples.

Samples	Regression Equations	R^2^	Linear Range (ng/mL)	LLOQ (ng/mL)
Plasma	Y = 0.129 × 10^−3^X − 0.0021	0.9967	10–5000	10
Heart	Y = 2.76 × 10^−3^X − 0.3078	0.9904	25–5000	25
Liver	Y = 2.76 × 10^−3^X − 0.3595	0.9978	25–5000	25
Spleen	Y = 3.01 × 10^−3^X − 0.0547	0.9943	25–5000	25
Lung	Y = 1.58 × 10^−3^X + 0.1471	0.9975	25–5000	25
Kidney	Y = 2.80 × 10^−3^X − 0.1467	0.9964	25–5000	25
Stomach	Y = 1.52 × 10^−3^X + 0.1601	0.9968	25–5000	25
Intestine	Y = 1.52 × 10^−3^X + 0.05300	0.9979	25–5000	25
Rat Liver Microsome	Y = 0.0959 × 10^−3^X + 0.1471	0.9910	50–50000	50

**Table 2:** Precision and accuracy of the determination of the analytes in rat biological samples (n = 6).

Samples	Analyte Concentration	Accuracy (%)	Intra-Day Precsion (%)	Inter-Day Precsion (%)
	(ng/mL)			
Plasma	10	−4.0	7.8	9.6
	25	−4.3	10	11
	300	3.2	9.7	8.5
	4000	3.9	9.0	9.7
Heart	25	4.2	8.1	10
	100	3.9	12	9.1
	600	−3.1	10	7.6
	4000	2.5	11	10
Liver	25	3.6	8.3	9.2
	100	4.5	11	11
	600	3.9	8.5	9.0
	4000	−3.5	9.5	8.8
Spleen	25	−4.1	8.6	10
	100	2.9	12	8.7
	600	−2.4	10	9.2
	4000	3.4	9.7	7.6
Lung	25	3.3	9.8	10
	100	3.1	12	9.4
	600	−2.6	7.2	10
	4000	3.2	10.0	6.6
Kidney	25	−4.6	11	11
	100	4.5	9.9	8.8
	600	−3.8	10	7.8
	4000	2.2	7.4	9.5
Stomach	25	4.3	10.2	8.4
	100	3.0	10	9.8
	600	2.5	5.1	7.0
	4000	3.3	8.3	7.5
Intestine	25	3.7	10	8.8
	100	3.8	7.6	11
	600	2.2	6.9	9.6
	4000	−3.0	9.2	7.1
Rat Liver Microsome	50	2.9	10	9.2
	250	3.0	6.2	7.2
	2000	2.6	7.7	8.1
	20000	2.2	7.4	7.2

**Table 3:** Matrix effects and extraction recovery for the analytes in rat biological samples (n = 6).

Samples	Spiked Concentration (ng/mL)	Matrix Effect		Extraction Recovery	
		Mean (%)	RSD (%)	Mean (%)	RSD (%)
Plasma	25	92.4	9.4	81.9	8.4
	300	96.5	6.3	88.0	9.8
	4000	102.5	8.6	82.9	7.4
Heart	100	88.9	10.9	85.0	9.7
	600	93.5	7.9	87.4	8.8
	4000	87.0	8.7	88.6	8.0
Liver	100	94.3	9.2	90.1	8.6
	600	94.5	8.9	86.3	7.9
	4000	87.7	10.0	81.2	8.3
Spleen	100	101.2	9.6	80.2	7.0
	600	105.1	7.3	79.6	8.5
	4000	94.3	9.0	82.0	9.7
Lung	100	88.0	8.0	81.4	6.7
	600	90.1	8.5	78.6	7.9
	4000	84.3	9.7	84.2	8.5
Kidney	100	104.6	9.4	87.6	9.2
	600	92.6	7.4	80.0	9.0
	4000	103.1	9.1	87.1	7.2
Stomach	100	98.6	8.2	84.3	6.4
	600	87.4	9.1	83.1	7.4
	4000	100.2	7.6	87.1	8.2
Intestine	100	98.5	10.0	79.0	9.7
	600	87.3	8.9	82.0	7.3
	4000	95.3	7.5	85.3	8.5
Rat Liver Microsome	250	91.5	7.8	81.3	8.0
	2000	91.0	8.3	87.1	8.2
	20000	90.3	8.6	82.3	7.7

**Table 4:** Stability data of the analytes in rat biological samples under different conditions (n = 6).

Samples	Spiked Concentration	Stability (% RE)			
	(ng/mL)	Three Freeze-Thaw	Short-Term	Long-Term	Post-Preparative
Plasma	25	4.3	−4.7	3.8	4.7
	300	−3.2	2.3	4.7	−4.4
	4000	4.4	4.7	−4.9	2.5
Heart	100	−6.2	3.0	4.0	4.4
	600	−5.3	4.8	3.0	−3.9
	4000	2.4	−4.4	3.2	4.7
Liver	100	−4.7	3.6	4.1	−4.0
	600	2.2	−4.3	3.9	4.2
	4000	3.3	4.2	3.1	4.8
Spleen	100	−3.2	2.7	-3.1	3.8
	600	4.5	3.5	2.7	−4.1
	4000	3.0	−4.0	4.3	−2.2
Lung	100	−3.1	2.8	3.6	3.6
	600	4.2	4.1	3.8	3.7
	4000	3.5	4.0	−2.9	4.4
Kidney	100	3.0	−2.7	3.2	4.9
	600	−2.8	3.1	4.1	−3.0
	4000	5.0	4.7	4.0	2.8
Stomach	100	3.2	−4.1	4.9	3.1
	600	−4.2	2.6	4.9	3.8
	4000	4.0	−3.9	−3.1	−4.1
Intestine	100	4.8	3.7	3.0	4.1
	600	4.7	−3.6	3.6	4.0
	4000	−3.5	3.1	3.0	4.5
Rat liver microsome	250	2.2	3.6	3.2	3.3
	2000	3.1	2.7	2.3	3.6
	20000	3.0	2.6	3.7	3.0

**Table 5:** Pharmacokinetics parameters of KM (10 mg/kg, intravenous administration) in rat that received or did not receive GU decoctions.

Parameters	Unit	KM Group	GU + KM Group
AUC_0–6_	μg/L * h	3340 ± 410	2410 ± 130 *
AUC_0–∞_	μg/L * h	3400 ± 420	2600 ± 200 *
MRT_0–t_	h	1.1 ± 0.1	1.3 ± 0.2
MRT_0–∞_	h	1.2 ± 0.1	1.8 ± 0.4
*t*_1/2z_	h	1.2 ± 0.1	1.7 ± 0.3 *
CL_Z_	L/h/kg	3.0 ± 0.4	3.9 ± 0.3 *
V_Z_	L/kg	5.0 ± 0.4	9.2 ± 0.9 **
*C*_max_	μg/L	5790 ± 410	3600 ± 1260 *

Data are expressed as mean ± SD. * *p* < 0.05, ** *p* < 0.01 compared with the KM group.

**Table 6:** *K_m_*, *V_max_,* and CL_int_ for koumine that received or did not receive GU decoctions in rat liver microsome (RLM).

Parameters	Unit	Control Group	GU-Pretreated Group
*K_m_*	μM	39.8 ± 23.3	69.9 ± 22.4
*V_max_*	nmol/min/mg protein	193.8 ± 96.6	419.6 ± 121.7 *
CL_int_	μL/min/mg protein	5.0 ± 0.4	6.0 ± 5.4

Data are expressed as mean ± SD. * *p* < 0.05 compared with the control group.

## Supplementary Materials

The following are available online at http://www.mdpi.com/1420-3049/23/7/1693/s1, Table S1: The extraction recovery for both LLE and PPM method. Table S2: The regression equations, linear ranges, LLOQs and uncertainty of coefficients of a and b for the determination of the analytes in rat biological samples.

## References

[1] Jin G.L., Su Y.P., Liu M., Xu Y., Yang J., Liao K.J., Yu C.X. (2014). Medicinal plants of the genus Gelsemium (Gelsemiaceae, Gentianales)—A review of their phytochemistry, pharmacology, toxicology and traditional use. J. Ethnopharmacol..

[2] Yuan Z.H., Liang Z.E., Wu J., Yi J.E., Chen X.J., Sun Z.L. (2016). A Potential Mechanism for the Anti-Apoptotic Property of Koumine Involving Mitochondrial Pathway in LPS-Mediated RAW 264.7 Macrophages. Molecules.

[3] Yuan Z., Matias F.B., Wu J., Liang Z., Sun Z. (2016). Koumine Attenuates Lipopolysaccaride-Stimulated Inflammation in RAW264.7 Macrophages, Coincidentally Associated with Inhibition of NF-kappaB, ERK and p38 Pathways. Int. J. Mol. Sci..

[4] Chen C.J., Zhong Z.F., Xin Z.M., Hong L.H., Su Y.P., Yu C.X. (2017). Koumine exhibits anxiolytic properties without inducing adverse neurological effects on functional observation battery, open-field and Vogel conflict tests in rodents. J. Nat. Med..

[5] Karwacki Z., Niewiadomski S., Rzaska M., Witkowska M. (2014). The effect of bispectral index monitoring on anaesthetic requirements in target-controlled infusion for lumbar microdiscectomy. Anaesthesiol. Intensive Ther..

[6] Rujjanawate C., Kanjanapothi D., Panthong A. (2003). Pharmacological effect and toxicity of alkaloids from Gelsemium elegans Benth. J. Ethnopharmacol..

[7] Gong H., Zhang B.K., Yan M., Fang P.F., Li H.D., Hu C.P., Yang Y., Cao P., Jiang P., Fan X.R. (2015). A protective mechanism of licorice (Glycyrrhiza uralensis): Isoliquiritigenin stimulates detoxification system via Nrf2 activation. J. Ethnopharmacol..

[8] Xiong B.J., Xu Y., Jin G.L., Liu M., Yang J., Yu C.X. (2017). Analgesic effects and pharmacologic mechanisms of the Gelsemium alkaloid koumine on a rat model of postoperative pain. Sci. Rep..

[9] Sun L., Lei L., Fang F., Yang S., Wang J. (1999). Inhibitory effects of koumine on splenocyte proliferation and humoral immune response in mice. Pharmacol. Clin. Chin. Mater. Med..

[10] Wang L., Wen Y., Meng F. (2018). Simultaneous determination of gelsemine and koumine in rat plasma by UPLC-MS/MS and application to pharmacokinetic study after oral administration of Gelsemium elegans Benth extract. Biomed. Chromatogr..

[11] Huang J., Su Y.P., Yu C.X., Xu Y., Yang J. (2010). Cytotoxic effects of alkaloidal compounds from Gelsemium elegans Benth on the tumor cells of digestive system in vitro. Strait Pharm. J..

[12] Hu Y., Wang Z., Huang X., Xia B., Tang L., Zheng Z., Ye L. (2017). Oxidative metabolism of koumine is mainly catalyzed by microsomal CYP3A4/3A5. Xenobiotica.

[13] Lowry O.H., Rosebrough N.J., Farr A.L., Randall R.J. (1951). Protein measurement with the Folin phenol reagent. J. Biol. Chem..

