# Supplementary material for: Ultra-Liquid Chromatography Tandem Mass Spectrometry (UPLC-MS/MS)-Based Pharmacokinetics and Tissue Distribution Study of Koumine and the Detoxification Mechanism of Glycyrrhiza uralensis Fisch on Gelsemium elegans Benth
Source: Molecules. 2018 Jul 11;23(7):1693. doi: 10.3390/molecules23071693 (PMC6100507; doi:10.3390/molecules23071693)
Supplement: Supplementary file 1 [file molecules-23-01693-s001.pdf]

molecules MDPI

Supporting information

Article

Ultra-Liquid Chromatography Tandem Mass Spectrometry (UPLC-MS/MS)-based Pharmacokinetics and Tissue Distribution Study of Koumine and the Detoxification Mechanism of *Glycyrrhiza uralensis* Fisch on *Gelsemium elegans* Benth.

Lin Wang, Qi Sun, Nan Zhao, Yan-qing Wen, Song Yang and Fan-hao Meng *

School of Pharmacy, China Medical University, 77 Puhe Road, Shenyang 110122, Liaoning, China; wanglin@cmu.edu.cn (L.W.); zgydsunqi@163.com (Q.S.); zhaonan-dd@163.com (N.Z.); cmu20038915@sina.com (Y.-Q.W.); Songyanglhyb1998@163.com (Y.S.)

* Correspondence: fhmeng@cmu.edu.cn; Tel.: +86-24-3193-9448

Received: 11 May 2018; Accepted: 2 July 2018; Published: date

S1. Optimization of Sample Preparation

The LLE method was explored for the six analytes by different types of solvents such as ethyl acetate, ether and dichloromethane. An aliquot of 10 μL IS solution was added to 100 μL biological matrix. 3 mL aliquot of extraction agent was added and mixture vortexed for 1 min. Then the samples were put into a centrifuge. The organic supernatant was pipetted into clean glass tubes individually after centrifugation 4000 rpm for 5 min. The upper organic layer was removed and evaporated to dryness at 40 °C under a steam of nitrogen. The residue was then reconstituted with 50 μL methanol, 5 μL of the sample solution was injected into the UPLC-MS/MS system for analysis. The recoveries for the LLE method were low. PPM, a simple and fast technique, has obtained satisfactory recoveries and reduced the endogenous-related substances in bio-matrix. The data of extraction recovery was shown in Table S1.

Table S1. The extraction recovery for both LLE and PPM method.

Samples	Spiked Concentration (ng/ml)	LLE-ether		LLE-dichloromethane		LLE-ethyl acetate		PPM	
		Mean (%)	RSD (%)	Mean (%)	RSD (%)	Mean (%)	RSD (%)	Mean (%)	RSD (%)
Plasma	25	49.6	9.8	58.1	7.1	62.3	9.6	81.9	8.4
	300	54.2	7.5	65.2	9.4	71.9	8.4	88	9.8
	4000	58.4	6.2	59.6	8.7	60.1	9.3	82.9	7.4

S2. Method validation

The specificity was assessed by analyzing 6 different matrices to probe no interference at the retention time of the analyte and IS from endogenous or exogenous components.

The calibration curve was determined by seven different concentrations of standard biological samples. The linearity of each calibration curve was conducted by the plot of the peak-area ratios (y) of the analytes to the IS against the nominal concentration (x) of calibration standards with weighted ($1/x^2$) least square linear regression. The LLOQ was defined as the lowest concentration of the calibration curve. The regression equation and uncertainty of coefficients of a and b for the determination of the analytes in rat biological samples.

Table S2. The regression equations, linear ranges, LLOQs and uncertainty of coefficients of a and b for the determination of the analytes in rat biological samples.

Samples	Regression Equations Y = aX + b	R^2	Linear Range (ng/mL)	LLOQ (ng/mL)	Ucertainty a	b
Plasma	$Y = 0.12939 \times 10^{-3}X - 0.0021$	0.9967	10–5000	10	5.6×10^{-4}	0.72×10^{-3}
Heart	$Y = 2.76 \times 10^{-3}X - 0.3078$	0.9904	25–5000	25	0.45×10^{-4}	9.4×10^{-3}
Liver	$Y = 2.76 \times 10^{-3}X - 0.3595$	0.9978	25–5000	25	0.18×10^{-4}	6.2×10^{-3}
Spleen	$Y = 3.01 \times 10^{-3}X - 0.0547$	0.9943	25–5000	25	0.29×10^{-4}	1.1×10^{-3}
Lung	$Y = 1.58 \times 10^{-3}X + 0.1471$	0.9975	25–5000	25	0.22×10^{-4}	5.1×10^{-3}
Kidney	$Y = 2.8 \times 10^{-3}X - 0.1467$	0.9964	25–5000	25	0.31×10^{-4}	6.5×10^{-3}
Stomach	$Y = 1.52 \times 10^{-3}X + 0.1601$	0.9968	25–5000	25	0.29×10^{-4}	4.8×10^{-3}
Intestine	$Y = 1.52 \times 10^{-3}X + 0.05300$	0.9979	25–5000	25	0.46×10^{-4}	9.1×10^{-3}
Rat Liver Microsome	$Y = 0.09588 \times 10^{-3}X + 0.1471$	0.991	50–50000	50	3.2×10^{-4}	4.3×10^{-3}

The precision and accuracy were estimated by analyzing QC samples in six replicates. Precisions were expressed as RSD required less than 15%, and the accuracy to be within ± 15%.

The matrix effects were measured by six independent sources of matrix. It was calculated by comparing the responses of the post extracted biological samples with that of pure standard solution containing equivalent amounts of the analytes. The extraction recovery was performed by comparing the peak area rations of KM to IS of an extracted sample to the standard analytes solution of the same concentration.

The stability of KM in biological samples was determined by analyzing six replicates of QC samples at four different manipulations involving short term stability (stored for 4 h at room temperature and kept at 4 °C for 24 h), long-term stability (stored for 2 weeks at −20 °C), freeze-thaw stability (−20 °C to room temperature as one cycle) and post-preparative stability (stored for 12 h after sample preparation at 4 °C).

Dilution integrity was conducted by spiking the blank biological samples with the known QC concentrations above the upper limit of quantitation. The biological samples were diluted with two fold. The acceptance accuracy and precision should not exceed 15%.
